# Maternal levels of endocrine disruptors, polybrominated diphenyl ethers, in early pregnancy are not associated with lower birth weight in the Canadian birth cohort GESTE

**DOI:** 10.1186/s12940-016-0134-z

**Published:** 2016-04-12

**Authors:** Yasmine K. Serme-Gbedo, Nadia Abdelouahab, Jean-Charles Pasquier, Alan A. Cohen, Larissa Takser

**Affiliations:** Department of Pediatrics, Faculty of Medicine, University of Sherbrooke, 3001 12e Avenue Nord, Sherbrooke, Quebec J1H 5N4 Canada; Department of Gynecology and Obstetrics, Faculty of Medicine, University of Sherbrooke, 3001, 12e Avenue Nord, Sherbrooke, Quebec J1H 5N4 Canada; Department of Family Medicine, PRIMUS Research Group, University of Sherbrooke, 3001, 12e Avenue Nord, Sherbrooke, Québec J1H 5N4 Canada

**Keywords:** Polybrominated diphenyl ethers, Pregnancy, Birth weight, Polychlorinated biphenyls, Mercury, Lead, Cadmium, Manganese

## Abstract

**Background:**

Polybrominated diphenyl ethers are known endocrine disrupting environmental contaminants used as flame retardants. Their levels have increased in humans over the last ten years, raising concerns about their consequences on human health. Some animal studies suggest that PBDEs can affect fetal growth; however, the results of human studies are contradictory. This study evaluates the association between the most common PBDEs in maternal blood measured in early pregnancy and birth weight.

**Methods:**

BDE-47, BDE-99, BDE-100 and BDE-153 levels were measured in 349 women during their first prenatal care visit at the University Hospital Center of Sherbrooke (Quebec, Canada). Birth weight and relevant medical information were collected from medical records. In contrast with previous studies, we examined the full range of clinical risk factors known to affect fetal growth as potential confounders, as well as other environmental pollutants that are likely to interact with fetal growth (polychlorinated biphenyls (PCBs), mercury, lead, cadmium and manganese).

**Results:**

There was no statistically significant relationship between PBDE levels in early pregnancy and birth weight in both unadjusted and multivariate regression models.

**Conclusions:**

Our results suggest that PBDEs in early pregnancy have little or no direct impact on birth weight, at least at the levels of exposure in our population.

**Electronic supplementary material:**

The online version of this article (doi:10.1186/s12940-016-0134-z) contains supplementary material, which is available to authorized users.

## Background

Polybrominated diphenyls ethers (PBDEs), are ubiquitous environmental contaminants widely used as flame-retardants in many consumer products (polyurethane foams used in furniture, mattresses, carpet pads and automobile seats and styrene plastics used for electrical appliances and flame-retardant textiles) [1]. PBDEs have been the focus of research over the past decade due to their ubiquitous presence in the environment [2, 3] and the human body (adipose tissue, blood, and breast milk) [3] in addition to their endocrine disrupting properties [4]. Furthermore, they are lipophilic compounds which easily cross the placental barrier.

Several reports, including ours [5, 6], indicate that PBDEs are thyroid disruptors in animals and humans, and a few studies suggest that PBDEs may potentially disrupt the insulin-like growth factor (IGF) system, which is an anabolic stimulus of fetal growth [7]. Indeed, uterine expression of insulin-like growth factor 1 (IGF-1) was upregulated in adult rat offspring exposed prenatally to 1 mg/(kg day) of BDE-99 [8]. Another study found that perinatal exposure to 0.002 – 0.2 mg BDE-47/kg increased body weight in all Wistar rat offspring, accompanied by increased plasma IGF-1 and glucose uptake in males (but not in females) [9]. To our knowledge, only two studies have examined the relationship between the IGF system and PBDEs in humans. The first study found a positive association between breast milk BDE-196 and IGF-1 in the cord blood from 149 women in the general Taiwanese population but they also found a negative association for BDE-99 and 86 [10]. The second study suggested a positive correlation between umbilical cord serum PBDE levels and placental gene expression of insulin-like growth factor binding protein 3 (IGFBP-3) among Chinese children living in one of the world’s largest electronic waste sites [11].

We hypothesized that, through their interaction with the IGF system, PBDEs could influence BW. Considering the role of IGF-1 and IGFBP-3 in the regulation of fetal growth [12], a positive association between prenatal exposure to PBDEs and these hormones may lead to a positive association between PBDEs and BW. However, among the nine epidemiological studies assessing the relation between PBDEs and BW (see Table 1), four studies reported a significant, negative association [13–16], two studies reported a non significant, negative association [17, 18], two others reported no statistically significant association [19, 20], and one study suggested a negative association for male infants and a positive association for female [21]. However, these epidemiological findings (i) were based on designs differing from each other and from our study, (ii) did not consider other ubiquitous environmental pollutants which could also affect fetal growth and (iii) did not take into account the relationship between BW and gestational age.

**Table 1:** Characteristics of 349 mothers-newborn in GESTE study, Quebec, Canada, 2007–2008

Characteristics (range and unit)	Mean (SD) No (%)
Total population	349 (100)
Newborn characteristics	
Male	179 (51.3)
Birth weight (1,555 to 4,705 g)	3,366.6 (504.1)
Low birth weight (BW < 2500 g)	16 (4.6)
Gestational age at recruitment (3.3 to 28.6 weeks of amenorrhea)	11.01 (2.9)
Gestational age at birth (30.3 to 42.3 weeks of amenorrhea)	39.3 (1.6)
Prematurity (Gestational age at birth < 37 weeks of amenorrhea)	23 (6.6)
Maternal characteristics	
Age at delivery (18 to 40 years)	28.8 (4.4)
Married or common-law relationship	323 (93)
BMI at recruitment (16.4 to 48.9 kg/m^2^)	25.9 (5.9)
Smoked during pregnancy	65 (19)
Alcohol consumption during pregnancy	19 (5.4)
Recreational drug consumption during pregnancy	5 (1.4)
Infection during pregnancy	32 (9)
Previous low birth weight (<2500 g) infant in multipara (*n* = 236)	28 (12)
Previous preterm infant in multipara (*n* = 236)	16 (7)
Inter pregnancy interval^a^	
Low (<18 months)	57 (24.3)
≥18 and ≤ 60 months	147 (62.5)
Large (>60 months)	31 (13.2)

*Abbreviation: SD* standard deviation

^a^Time interval between pregnancy before and GESTE pregnancy

The present study examines the association between PBDEs in early pregnancy and BW in our prospective birth cohort, GESTE (*GEStation Thyroid and Environment*), which has been specifically designed to study health outcomes related to PBDE exposure in pregnant women and their children. We focus on the most prevalent PBDE congeners in maternal and umbilical-cord blood, which are BDE-47, BDE-99, BDE-100, and BDE-153 [14, 22–24]. Because PCBs are suspected to be associated with BW (particularly CB-153, which was consistently shown to be negatively associated with BW [21]), we analyzed the most prevalent PCBs in our samples as potential confounding factors, as well as metals (mercury, lead, cadmium, and manganese), which are also ubiquitous in the environment and have been reported to have an impact on fetal growth [25–30].

## Methods

### Population recruitment

In the GESTE cohort, we recruited pregnant women at their first prenatal visit (mean gestational age = 12 weeks, SD = 2.96 weeks). As previously described [6], 400 eligible women who agreed to participate at their first prenatal care visit at the Research Center of the CHUS (*Centre Hospitalier Universitaire de Sherbrooke*) in Quebec, Canada, between September 2007 and December 2008, were included. Virtually all pregnant women living in the region served by the CHUS are seen in early pregnancy for routine clinical tests including blood and urine sampling. Eligibility criteria were maternal age (≥18 years), gestational age < 20 weeks, and no known thyroid disease. Given our intention to follow children from recruited pregnancies, we excluded women who planned to move out of the region in the following three years. Gestational age was confirmed by ultrasonography. For this paper, four pregnancies for which gestational age was found to be > 20 weeks based on ultrasonography were kept in our analyses. We estimate the rate of refusal to participate to be less than 5 % for eligible women approached by members of the research team. Maternal blood was collected at recruitment in 10-mL Vacutainer Hemogard tubes with ethylenediaminetetraacetic acid (Becton-Dickinson, San Jose, California). The plasma was frozen at −20 °C in decontaminated Supelco glass storage tubes (Supelco, Inc., Bellefonte, Pennsylvania) for PBDEs, PCBs and thyroid hormone dosing. Blood was also obtained in 10-mL metal-free blood collection tubes with 0.05 mL of 15 % ethylenediaminetetraacetic acid K3 and stored at 4 °C for manganese, lead, mercury and cadmium analysis.

The study protocol was approved by the Human Research Ethics Committee of the CHUS, and an informed consent form was signed by each participant.

### Data collection

#### Outcome measurement

Information on BW was obtained from medical records completed at delivery by the obstetrical team who weighed newborns with a Scale-tronix pediatrics scale 4802.

#### Laboratory analysis

##### PBDE and PCB levels

Plasma levels of PBDEs (BDE-47, BDE-99, BDE-100, and BDE-153) and PCBs (CB-138, CB-153, and CB-180) were analyzed in our laboratory based on the method described by Covaci and Voorspoels [31].

Quality control was conducted through regular analyses of water blanks added at every 10th sample, solvent blanks, and random duplicate samples. All blanks were subtracted from sample values on a batch basis. Interbatch coefficients of variation were ≤10 % and ≤8 % for PBDEs and PCBs, respectively. Limits of detection (LOD), defined as 3 times the noise level, were established at 0.1 pg/μL for PBDEs and at 0.02 pg/μL for PCBs. Routine checks of accuracy and precision were also performed using reference materials (1589a) from the National Institute of Standards and Technology (Gaithersburg, MD, USA). Recoveries ranged between 75 and 125 %. Here, we used the sums of PBDE and PCB levels across classes, as well as the levels of individual PBDEs and PCBs.

##### Metal levels

Whole blood manganese, lead, cadmium and mercury level determinations were performed by the Toxicology Center of Quebec at the Quebec Institute for Public Health (CTQ-INSPQ). Cold vapor atomic absorption spectrophotometry was used for total blood mercury and mass spectrometry plasma torch (ICP-MS) for lead, cadmium and manganese.

Limits of detection were 2.07 μg/L for lead, 0.1 μg/L for mercury, 0.05 μg/L for cadmium, and 4.4 μg/L for manganese.

#### Other relevant information

Following inclusion, women answered a questionnaire administered by a research nurse who also measured women’s height and weight. The questionnaire was used to obtain women’s birth dates, education, marital status, income, obstetrical history, smoking habits, consumption of alcohol and recreational drugs. Another questionnaire was administered after delivery by phone to complete data for the period between recruitment and delivery. Pregnancy follow-up data (infection during pregnancy, BW and baby sex) were also extracted from medical records and gestational ages at recruitment and birth were calculated from the ultrasonography (see Additional file 1: Table S1 for details).

### Statistical analysis

All analyses were performed with SAS/STAT software (SAS version 9.3) [32]. PBDE levels were analyzed in 386 of 400 participants due to the loss of 11 samples as a result of analytical problems and the exclusion of twin pregnancies (*n* = 3) post-hoc, for a total of 14 samples. Additional losses to follow-up included 27 women with miscarriages, 5 with voluntary abortions and 5 who delivered in another hospital. Our analyses were therefore run on 349 newborn-mother pairs.

Analyses were conducted as follows: 1) Descriptive statistics on exposure data, BW, and potential confounding factors or modifiers were calculated. 2) Bi-variate Spearman correlation analysis was performed to determine potential colinearities between contaminants, BW and blood sampling time (gestational age at recruitment). 3) Simple and multivariate linear regressions were carried out to study the association between BW and PBDEs.

A major challenge for a study of this nature is the lack of empirical or biological basis for choosing among an excessively large number of potential model specifications. In particular, covariates and confounders are problematic, given that many potentially important variables have not been measured in previous studies, and that the evidence on causal pathways necessary for constructing a directed acyclic graph is largely absent. Additionally, BW and gestational age are closely, non-linearly associated, and we are not able to confirm if PBDEs could act on either gestational duration as suggested in one preceding study [33] or on growth rates, creating a collider bias in estimating the association between PBDE and BW if adjusted on gestational age [34]. In addition, given that lipophilic pollutants, such as PCBs (and PBDEs) are bound to blood lipids [35, 36], the metabolism of which is rapidly changing in early pregnancy [37], we need to consider different types of multivariate modeling to take into account possible causal pathways.

To find the most relevant multiple regression models, we first tested the relationship between BW and (i) other pollutants (PCBs, cadmium, lead, mercury, manganese), (ii) potential confounders or modifiers, which have been extensively reviewed and shown to be moderately or strongly associated with low BW by the Institute of Health Economics (IHE) [38]. These potential confounders were: gestational age at birth, mother’s age at delivery, marital status (single or not), Body Mass Index (BMI) at recruitment, infection, smoking status, alcohol and recreational drug consumption during pregnancy, and inter-pregnancy interval. We have also tested infant sex and gestational age at recruitment as potential covariates [39]. Since a previous study found a different association between PBDEs and IGF-1 according to sex [9], we also tested the statistical interaction between sex and PBDE levels. Since this interaction was not significant, these data are not shown.

Our initial models were simple linear regression between PBDEs and BW. Covariates were included in multiple linear regression models if they were associated in bivariate analyses with one PBDE congener or the sum of PBDE and BW at *P* < 0.25. The following variables were included in final multivariate models: mother’s age, marital status, BMI, infections, smoking, PCBs, and lead levels. We used the sum of PCBs (CB-138, 153, 180) in our analyses as they were inter-correlated (Table 3).

#### Dependent variable

Gestational age was the most correlated with BW (*r* = 0.53, *p* < 0.001). To avoid collider bias [34], we created a dependent variable “BW corrected for gestational age”, which was used in all subsequent statistical models. The correction of BW was done using residues from non-parametric regression “SAS PROC LOESS” with 0.75 as the smoothing parameter (see Additional file 1: Figure S1), as follows:



We choose this procedure because the inter-quartile interval of residues in this model was smaller compared with linear, spline or curviline regression. The obtained residuals were normally distributed (*p*-value for Kolmogorov-Smirnov > 0.25).

#### Independent variable related to PBDE exposure

Among the 349 samples, 5 %, 23 % and 21 % (Table 2) were below the limit of detection (LOD), which were for BDE-99 (LOD = 0.001 ng/mL), BDE-100 (LOD = 0.001 ng/mL) and BDE-153 (LOD = 0.0006 ng/mL), respectively. We ran linear regression analysis using PBDEs congener individually by replacing below-LOD values by LOD/2. To create the summed values (ΣPBDEs and ΣPCBs), values below the limit of detection we used zero for undetectable congeners.

**Table 2:** Levels of PBDEs, PCBs, selenium, mercury, lead, cadmium and manganese in early pregnancy (*N* = 349)

	% of detected values	25^th^ percentile	50^th^ percentile	75^th^ percentile	Maximum
PBDE congeners (ng/mL)					
PBDE-47	100	0.07	0.12	0.25	2.74
PBDE-99	95	5.10^−3^	0.01	0.03	0.48
PBDE-100	77	4.10^−3^	7.10^−3^	0.01	0.36
PBDE-153	79	8.10^−3^	0.02	0.05	0.39
Σ PBDEs		0.10	0.19	0.34	3.63
PBDE congeners (ng/g of lipids)					
PBDE-47		10.78	21.23	40.82	548.00
PBDE-99		0.76	1.93	5.44	96.00
PBDE-100		0.66	1.32	2.44	81.63
PBDE-153		1.08	2.44	8.97	85.33
Σ PBDEs		17.08	32.99	59.63	726.00
PCBs (ng/g of lipid)^a^					
PCB-138	93	2.29	4.25	10.41	178.04
PCB-153	99	4.55	8.02	19.80	410.43
PCB-180	100	2.02	4.11	9.02	90.91
Σ PCBs		10.91	18.87	42.80	455.78
Metals^b^					
Manganese (ng/mL)	100	7.70	9.35	11.55	32.45
Mercury (ng/mL)	85	0.38	0.62	1.01	6.83
Lead (ng/mL)	100	6.21	8.28	10.35	64.17
Cadmium (ng/mL)	100	0.11	0.22	0.36	6.04

*Abbreviations: PBDE(s)* polybrominated diphenyl ether(s), *ΣPBDE* total PBDEs, *PCB(s)* polychlorinated biphenyl(s), *ΣPCBs* total PCBs

^a^Missing values for PCBs, *n* = 2

^b^Missing value for at least one metal (*n* = 57)

Each PBDE congener was introduced in regression models separately to avoid potential colinearity. In addition, we used the sum of PBDEs as commonly used in the literature in order to compare our results with previous studies. We have verified that for all models, residuals were normally distributed (*p*-value for Kolmogorov-Smirnov test > 5 %).

For each congener, we ran both: (i) volume-based models where total plasma lipid levels were introduced as an independent variable, and (ii) lipid-based models where PBDEs were expressed in ng/g lipids.

Underlying distributions of PBDE congeners appeared to be log-normal, so log_10_ transformation improved normality. Thus, we ran regression models using (i) PBDEs non transformed concentrations and (ii) log_10_-transformed PBDEs. In the results section, we only present the results with PBDEs log_10_-transformed. Results with non transformed PBDEs are presented in Additional file 1: Table S3.

## Results

Socio-demographic information, lifestyle habits, BMI at recruitment, obstetric history and pregnancy outcomes are presented in Table 1. The majority of women were married or in a common-law relationship (93 %) and 68 % were multiparous. Among multiparous women 12 % and 7 % had a history of low birth weight or premature delivery, respectively. The time interval between their last delivery and the current pregnancy ranged between 18 and 60 months for 63 %, less than 18 months for 24 % and more than 60 months for 13 % of multiparous women. Regarding mothers’ lifestyle factors, 19 % reported being active cigarette smokers (the number of cigarettes per day ranged from one to 20 in the first trimester and from one to 15 in both the second and third trimesters), 5 % to having consumed alcohol at least once and 1 % to having used recreational drugs during pregnancy. At recruitment, the women ranged from 18 to 40 years old with an average age of 28 years. Their BMI was between 16 to 49 Kg/m^2^ at inclusion (average of 26 Kg/m^2^). Fifty-one percent of babies were male, sixteen babies (4.6 %) weighed less than 2500 g at birth and 23 (6.6 %) were born prematurely (gestational age < 37 weeks).

PBDE, PCB and metal levels are shown in Table 2 and their inter-correlations are shown in Table 3. CB-138, 153 and 180 were detected respectively in 93 %, 99 % and 100 % of the samples. PBDE levels were inter-correlated among congeners, but not all correlations were strong, and were generally weaker than among PCB congeners. Lead, cadmium and manganese were detected in all maternal samples. Manganese levels ranged between 4.4 and 32.45 μg/L. Mercury was detected in 85 % of cases in concentrations ranging from 0.1 to 6.8 μg/L. Among metals, inter-correlations ranged between −0.07 and 0.31.

**Table 3:** Spearman correlation coefficients for PBDEs, PCBs, mercury, lead, cadmium, manganese levels, birth weight and gestational age at recruitment (*N* = 349)

	BW	GAR	Cadmium	Manganese	Mercury	Lead	Sum of PBDEs	BDE-47	BDE-99	BDE-100	BDE-153	Sum of PCBs	CB-138	CB-153
BW	-													
GAR	−0.03	-												
Cadmium	−0.12*	0.02	-											
Manganese	0.05	−0.06	−0.07	-										
HgT	−0.01	−0.02	0.07	0.31***	-									
Lead	−0.10	−0.01	0.24***	0.25***	0.17**	-								
Sum of PBDEs	0.03	0.05	−0.03	0.00	0.04	−0.02	-							
BDE-47	0.05	0.08	−0.03	−0.01	0.04	−0.04	0.95***	-						
BDE-99	0.06	−0.004	−0.02	0.00	0.01	−0.03	0.73***	0.58***	-					
BDE-100	0.02	0.06	−0.04	0.03	0.00	0.06	0.28***	0.11	0.19**	-				
BDE-153	−0.09	−0.09	0.01	0.01	0.00	0.10	0.53***	0.32***	0.54***	0.14*	-			
Sum of PCBs	0.08	0.12*	−0.08	0.12*	0.06	0.08	0.26***	0.26***	0.22***	0.09	0.12*	-		
CB-138	0.10*	0.10*	−0.05	0.08	0.03	0.04	0.12*	0.13*	0.08	0.09	0.06	0.76***	-	
CB-153	0.05	0.09	−0.06	0.14*	0.06	0.05	0.31***	0.32***	0.25***	0.06***	0.09	0.84***	0.39	-
CB-180	0.046	0.09	−0.06	0.03	0.04	0.12*	0.13*	0.12*	0.17**	0.06	0.17**	0.75***	0.49***	0.46***

All contaminants were expressed in ng/mL and below-LOD values were excluded. BW is expressed in g

*GAR* Gestational age at recruitment

* < 0.05; ** < 0.01; *** < 0.0001

The results of simple linear regression between BW and potential confounders are detailed in Additional file 1: Table S2. Gestational age at birth, sex, infection, smoking during pregnancy and maternal pre-pregnancy BMI, maternal blood levels of cadmium were significantly associated with BW at *p* < 0.05. In these relationships, gestational age at birth explained 28 % and sex 2 % of BW variance. The variance explained by other confounders ranged from 0.4 % for inter-pregnancy interval to 2 % for maternal BMI at recruitment and maternal infection during pregnancy. Early pregnancy levels of cadmium explained 1 % and PCBs explained 0.8 % of BW variance, respectively.

Table 4 shows the results of linear regression models for PBDEs (each congener and the sum) in early pregnancy (expressed in ng/g of lipid and log_10_-transformed) in relation to BW corrected for gestational age. PBDEs were not significantly associated with BW corrected for gestational age in both adjusted and unadjusted linear regression. Furthermore, all these associations remained non-significant in linear regressions adjusted for confounding factors. Interactions between sex and PBDEs were never statistically significant (data not shown).

**Table 4:** Association between birth weight corrected for gestational age and maternal plasma levels of PBDEs (log_10_) in early pregnancy

Log_10_ PBDEs	Simple linear regressions^a^		Multivariate linear regression^b^			
			Model A		Model B	
	β	95 % CI	β	95 % CI	β	95 % CI
Log_10_ PBDEs (ng/g of lipid)						
BDE-47	0.8	−93.1; 94.7	−25.1	−120.4; 70.3	−14.4	−129.9; 101.2
BDE-99	33.7	−34.8; 102.2	20.6	−49.1; 90.4	−1.6	−90.9; 87.7
BDE-100	25.4	−32.5; 83.3	23.9	−33.6; 81.5	4.7	−67.2; 76.5
BDE-153	−49.6	−100.9; 1.7	−40.7	−91.9; 10.6	−55.9	−118.9; 6.9
ΣPBDEs	−25.4	−128.7; 77.9	−46.1	−151.3; 59.1	−44.7	−173.6; 84.1
Log_10_ PBDEs (ng/ml)^c^						
BDE-47	13.6	−82.7; 109.9	−16.1	−113.5; 81.3	3.0	−115.9; 121.9
BDE-99	46.7	−27.1; 120.5	30.1	−45.2; 105.4	10.2	−86.7; 107.1
BDE-100	30.7	−27.8; 89.2	27.9	−30.1; 85.9	11.6	−61.0; 84.2
BDE-153	−49.7	−103.1; 3.7	−39.6	−103.0; 23.8	−55.0	−120.6; 10.6
ΣPBDEs	−12.3	−123.2; 98.5	−39.2	−151.7; 73.3	−27.0	−166.1; 112.0
Log_10_ PBDEs (ng/ml) adjusted for total plasma lipids.						
BDE-47	14.5	−81.8; 110.8	−16.2	−113.6; 81.2	6.5	−112.3; 125.4
BDE-99	70.4	−8.6; 149.4	48.4	−32.3; 129.2	42.1	−63.7; 147.9
BDE-100	31.1	−27.4; 89.6	27.9	−30.1; 85.8	12.8	−59.7; 85.4
BDE-153	−45.4	−100.4; 9.6	−37.5	−92.2; 17.2	−47.1	−115.3; 21.1
ΣPBDEs	−2.6	−114.9; 109.7	−32.8	−146.5; 80.9	−9.3	−151.3; 132.6

*Abbreviations: CI* confidence interval, *PBDE(s)* Polybrominated diphenyl ether(s), *ΣPBDE* sum of BDE-47, 99, 100 and 153

^a^Non-adjusted for potential confounders

^b^Adjusted for potential confounders

Model A: Adjusted on mother’s age at delivery, marital status, BMI at recruitment, infection, smoking status during pregnancy, ΣPCBs (CB-138, CB-153 and CB-180 expressed in ng/g of lipid), mothers total blood levels of lead (ng/ml)

Model B: Model A + previous pregnancies history (preterm infant and low birth weight infant) in multipara only (*N* = 234)

^c^Total lipids were not taken into account in these models

In sensitivity analyses we excluded neonates born before the 37^th^ week of gestation but the model estimates did not change substantially. We also conducted the same analyzes excluding subjects with BDE-99, BDE-100, or BDE-153 below LOD, but did not observe any change in the results.

## Discussion

We found no statistically significant association between BW and levels of BDE-47, BDE-99, BDE-100, BDE-153 and their sum. The absolute values of the slopes in regressions vary between 0.8 and 70.4 g of BW, suggesting a tiny clinical effect for a 10 fold increase in the concentration of PBDEs. These findings remained unchanged in both volume-based and lipid-based models.

Our study has the following methodological strengths (i) a large sample size, (ii) extensive measurement of known clinical and lifestyle risk factors for intrauterine growth restriction and measured levels of other environmental exposures (cadmium, manganese, lead, PBCs, and mercury), which have been reported to be negatively correlated with BW in the general population [25–30], (iii) replication of results across a wide range of modeling approaches, given the weak a priori knowledge available to support model construction; and (iv) the use of BW independently of its association with gestational age.

One of potential limitations of our study is that two PBDE congeners (BDE-100 and BDE-153) were undetectable in 23 % and 21 % of the samples, respectively. In our statistical analyses we considered them as LOD/2, which might induce an under or overestimation of regression slopes. However, we conducted sensitivity analyses, in which we excluded undetectable data and we obtained similar results.

Our population is representative of Canadian pregnant women for blood metal levels, maternal age and birth weight [40]. However, a greater proportion of the pregnant women in our study were smokers (19 %) compared to Canadian pregnant women (15 %) [41]. Alcohol consumption reported by the pregnant women in our study group (5 %) is lower than in Canada (15 %) and significantly lower compared to the rest of the Quebec province (26 %) [42]. High education levels in our study group was higher compared to pregnant women in Canada [42], which could be partly related to our inclusion criteria as well to the specificity of our recruitment area (Sherbrooke has two Universities and two regional Colleges). Median family income (CAD$60,000) was similar compared to women in Quebec in 2009 (CAD$64,000) [42]. An additional difference consists of the fact that the absolute majority of pregnant women were Caucasian (of French or Irish origin), which reflects an historical particularity of our area. Globally, our study group was more educated and more genetically homogenous, but similar to Canadians in terms of environmental exposures and birth weight. We believe that the high homogeneity of our study group represents an advantage for testing the effects of environmental exposures, because this situation is closer to a quasi-experimental design.

In Table 5, we summarized all available epidemiological data (9 publications) on PBDEs and BW. Four of them reported a negative association between PBDE levels in mothers and BW [13–16]. Harley et al. [13] found a significant negative correlation between PBDE levels in the second trimester and BW among 289 low income pregnant women who were also highly exposed to pesticides in California. In another study in Ontario, Canada [14], only BDE-99 in cord blood was negatively correlated with BW. In a Swedish cross-sectional study of 254 mother-babies, PBDE levels in breast milk were associated with a decrease in BW specifically in male infants [16]. In Spain, Lopez-Espinosa et al. reported a negative significant association between BDE-99 and BW [15]. In other five studies, there was no association [19, 20], statistically not significant association [18], or a positive association in female only [21], or the number of subjects was too small to conclude (*n* = 20, [17]).

**Table 5:** Medians of PBDE levels (ng/g lipid) in studies which reported the association between birth weight and PBDEs

Country (ref)	N	Year (s)	Specimen analyzed	BDE-47	BDE-99	BDE-100	BDE-153	Covariates	Association birth weight/PBDEs^a^
Sweden (24)	254	1996 - 2010	Breast milk	1.30	0.26	0.25	0.54	*Di-ortho* PCB, gestational length, moternal age, pre-pregnancy BMI, weight gain during pregnancy, education, smoking and infant sex.	Negative
California, USA (21)	286	1999 - 2000	Maternal serum (2^nd^ trimester of pregnancy)	14.57	3.85	2.45	2.03	Moternal age, marital status, parity, body mass index, country of birth (United States vs. other), family income, sex of infant	Negative
Taiwan^a^ (25)	20	2000 - 2001	Breast milk	1.52	0.51	0.37	0.87	Maternal age, pre-pregnancy BMI and parity.	BDE-47 geometric mean was higher for birth weight ≤ 3.05 Kg compared to birth weight > 3.05 Kg
Indiana, USA (5)	15	2001	Maternal serum (delivery)	28.00	5.70	4.20	2.90	-	No association
Ontario, Canada (22)	97	2004 - 2005	Maternal serum (24–28 WOA)	24.20	8.73	3.25	4.15	BMI, maternal age, parity, gestational age at birth, socio-economic status (family income and years of education), sex of the baby, and serum cotinine concentration.	Negative only for BDE-99
Australia (26)	150	2008 - 2011	Maternal plasma (38 WOA)	3.96	0.88	0.98	2.26	Maternal age, number of children, Gestational age, gender, Maternal BMI prior to pregnancy, maternal weight gain.	Negative not statistically significant
China (28)	215	2010 - 2012	Maternal serum (during pregnancy)	2.26	3.58	2.13	4.87	Maternal age, parity, education, pre-pregnancy BMI, weight gain during pregnancy, family income, gestational age	Positive association with PBDE-153 (in female infants only)
Spain (23)	473	2003 - 2008	Maternal serum (10–13 WOA)	< LOD	1.14	-	2.19		Negative only for BDE-99
	486		Umbilical cord blood	1.73	1.05	-	1.91	-	
North Carolina, USA (27)	137	2005 - 2008	Maternal serum (35–36 WOA)	18.87	5.5	4.61	5.65	Final models not show	No association
Our study	349	2007 - 2008	Maternal serum (<29 WOA)	20.93	4.03	2.21	4.21	Maternal age, marital status, BMI, infection, smoking status, PCBs, lead (ng/ml), plus previous pregnancies history (preterm infant and low birth weight infant) in multipara only (*N* = 234).	No association

^a^Mean

*WOA* Weeks of amenorrhea

The discrepancy among the results may be explained by the different designs of the studies, exposures levels, statistical analyses, adjustments methods, but also by the presence of underlying predisposing or susceptibility risk factors in a given population. An important aspect is that BW is a proxy for *in utero* growth, a multi-factorial phenomenon. Indeed, BW is influenced by multiple factors. These factors do not all have the same degree of influence and can interact synergistically or not, giving a multitude of possibilities [43]. This implies that the link between PBDEs and BW in two homogeneous (socially and/or genetically) populations with two different combinations of BW-related factors may not be the same. This theory was well explained by Rothman in 2005 [44]. He suggests that in multifactorial conditions, several causal components act in concert. A condition can be caused by different sets of combinations in different individuals or populations. Thus, it is possible that in our population, PBDEs have little or no role in determining BW, given the roles of genetic background, social environment, and complex interactions among these factors. Thus, our negative finding cannot exclude that PBDEs can affect populations with different social and/or genetic backgrounds.

For example, PBDE levels in the Californian study mentioned above were similar to those in our population [13] (Table 5). This study reported a negative association between PBDE levels in mid-pregnancy and BW in pregnant women from prenatal care clinics serving low-income residents who qualified for low-income health insurance (Medicaid). The majority (95 %) are Hispanic, while in our study, pregnant women were predominantly Caucasian Canadians (96.5 %) and had substantially higher socio-economic status (median family income was CAD$60,000). Because BW is strongly linked to lifestyle (nutritional status, social stress and maternal health), this difference between these two populations could explain the mismatch of our results, either through a synergistic relation between socioeconomic status and PBDEs, or through residual confounding. This emphasizes the importance of choosing a pertinent set of adjustment variables to assess the relationship between BW and PBDEs.

The Ontario study [14] reported a statistically significant negative correlation between BDE-99 and BW in a group of 97 women. Their study group was also mainly Caucasian (87 %), but older compared to ours (33 versus 29 years), and much more educated (84.5 % reported obtaining a university degree or greater versus 33 % in our data) and fewer were cigarette smokers (7 % versus 19 % in our population). The median levels of three PBDEs (−47, −100, and −153) in the Ontario study were similar to those in our population. However, the median level of congener BDE-99, for which they report a negative correlation with BW, is two-fold higher than in our study group. Although this may be due to inter-laboratory differences in analyses, but we cannot exclude that the Ontario study has a different exposure profile. Another limitation of the Ontario study is the small size of the study group and thus a limited statistical power to reliably affirm the decrease of 2 grams of BW per one ng/g lipids of BDE-99.

Lignell et al. [16] also found a significant negative relation between BW and the sum of PBDEs levels (BDE-47, BDE-99, BDE-100, BDE-153) measured in breast milk from 254 mothers in Sweden. These women were relatively close to our study group in terms of maternal age and education. They report that the association between PBDEs and BW was significant only after adjustment for di-ortho-PCBs (the same we considered in our analyses). However, it is important to note that the design of their study including retrospective exposure assessment (PBDEs in breast milk 3 weeks after delivery) as well as very low levels of PBDEs and narrow range of exposures (for example, the maximum level of BDE-153 in breast milk in our population was 180 ng/g lipids [16] versus 4.7 ng/g lipids in Lignell et al.’ study) are significant limitations for the comparison with our study.

In the Spanish study [15], which was one of the largest among available studies, only one PBDE congener (BDE-99) was significantly and negatively correlated with BW. The study group of the Spanish study was very different from our study group in terms of smoking rate (32 % versus 19 %), education level (24 % with primary educational level versus 3 % in our study), and PBDE exposure (high proportion of values below LOD). Although they reported that their database contains a large number of variables which are potential confounders, we could not identify to which confounders they adjusted their final models. In addition, it is not clear at which moment of pregnancy blood was taken for PBDE levels. This missing information limits the comparison with our study.

Interestingly, we recently reported a negative correlation between PBDE levels in early pregnancy and thyroxin levels in cord blood within the same population [6]. However, BW was not correlated with thyroid hormones measured in early pregnancy or in cord blood (data not shown) and the inclusion of thyroid hormones in our models did not change any statistical relationship between PBDEs and BW (data not shown). Thus, the lack of association between PBDEs and BW should not be construed as suggesting that PBDEs are safe during fetal development. Indeed, several human and experimental studies suggest a causal link between prenatal exposure to PBDEs and attention deficit [45]. Even if several contaminants, including endocrine disrupting chemicals, have been reported to be correlated with BW [13, 14, 16, 25–30], BW is a weakly sensitive or reliable proxy for developmental toxicity of environmental pollutants in humans [43], as shown in our low risk population. Conversely, PBDEs may affect brain development without any association with BW by disrupting thyroid hormones during the fetal period.

## Conclusions

Our prospectively collected data indicates that the link between PBDEs and BW does not exist, is very weak, or depends on the presence of other risk factors. However, this does not exclude the possibility of developmental PBDE toxicity via endocrine disruption.

## Additional file

Additional file 1: Table S1. Description of adjustment variables. **Table S2.** Simple linear regression between birth weight and potential confounding factors. **Table S3.** Linear relations between birth weight corrected for gestational age and mothers’ plasma levels of PBDEs (non-transformed) in early pregnancy (*N* = 349). **Figure S1.** Relation between predicted BW (obtained with proc LOESS) and gestational age at birth. (DOCX 84 kb)

## Abbreviations

BMI: Body Mass Index
BW: birth weight
CAD$: Canadian dollar
CHUS: Centre Hospitalier Universitaire de Sherbrooke
GESTE: Grossesse et Enfant en Santé-Etude sur la Thyroïde et l’Environnement
IGF: insulin-like growth factor
IGF-1: insulin-like growth factor 1
IHE: Institute of Health Economics Alberta, Canada
IUGR: intrauterine growth restriction
LOD: limit of detection
PBDE(s): polybrominated diphenyls ethers (s)
PCBs: polychlorinated biphenyls
WOA: weeks of amenorrhea
