# Supplementary material for: Maternal levels of endocrine disruptors, polybrominated diphenyl ethers, in early pregnancy are not associated with lower birth weight in the Canadian birth cohort GESTE
Source: Environ Health. 2016 Apr 12;15:49. doi: 10.1186/s12940-016-0134-z (PMC4828807; doi:10.1186/s12940-016-0134-z)
Supplement: Additional file 1: Table S1. — Description of adjustment variables. Table S2. Simple linear regression between birth weight and potential confounding factors. Table S3. Linear relations between birth weight corrected for gestational age and mothers’ plasma levels of PBDEs (non-transformed) in early pregnancy (N = 349). Figure S1. Relation between predicted BW (obtained with proc LOESS) and gestational age at birth. (DOCX 84 kb) [file 12940_2016_134_MOESM1_ESM.docx]

Additional file 1

Table S1. Description of adjustment variables

| **Variables** | | **Type** | **Units or coding** | **Collection, Questions wording, creation method** |
| --- | --- | --- | --- | --- |
| **Newborn characteristics** | |  |  |  |
| Gestational age at birth | | **Numeric** | **weeks** | Extracted from medical records (difference between gestational age at the first ultrasonography and the birth date) |
| Age at delivery | | Numeric | years | Questionnaire at enrolment (mother birth date), medical records (delivery date). |
| Newborn sex | | Binary | 0.Female  1.Male |  |
| **Women characteristics** | |  |  |  |
| Gestational age at recruitment | |  |  | Extracted from medical records (difference between gestational age at the first ultrasonography and the recruitment date) |
| Previous low birth weight (< 2500g) infant | | Binary | Yes or No | Extracted from women obstetrical file  Extracted from women obstetrical file |
| Previous preterm infant | | Binary | Yes or No |  |
| Inter pregnancy interval | | Categorical | 1. Low < 18 months  2. Intermediary ≥ 18 and ≤ 60 months  3. Large > 60 months | Represent the time between the end of the last pregnancy and the beginning of the pregnancy followed in GESTE. It was calculated by taking the end date of the previous pregnancy (delivery, abortion or miscarriage) mentioned in medical files and the starting date of the pregnancy GESTE (calculate from the first ultrasonography). The cut off was conventionally extracted from a meta-analysis of 67 Articles*. |
| Marital status | | Binary | 1. Married or common-law relationship  2. Single (single-parent, divorced or widowed) | Questionnaire at enrolment |
| BMI at recruitment | | Numeric | kg/m^2^ | Weight and height were measured at enrollment |
| ***During pregnancy*** | | | | |
| Smoking status | Binary | | Yes or No | Questionnaires at enrollment and after delivery |
| Alcohol consumption | Binary | | Yes or No |  |
| Recreational drugs | Binary | | Yes or No |  |
| Infection | Binary | | Yes or No | Any notification of infection and/or antibiotic therapy during pregnancy in medical record. |

* Conde-Agudelo A, Rosas-Bermúdez A, Kafury-Goeta AC. Birth spacing and risk of adverse perinatal outcomes: a meta-analysis. JAMA. 2006 Apr 19; 295(15):1809-23.

Table S2. Simple linear regression between birth weight and potential confounding factors

| **Characteristics** | **β_1_** | **95% CI** | **R^2^** |
| --- | --- | --- | --- |
| **Newborn characteristics** |  |  |  |
| Gestational age at birth (weeks of amenorrhea) | 165.5 | 137.5, 193.4*** | 0.28 |
| Male | 137.8 | 32.4, 243.1** | 0.02 |
| **Women characteristics**  Gestational age at recruitment (weeks of amenorrhea) | -5.8 | -23.7, 12.5 | 0.00 |
| Age at delivery (years) | 11.8 | -0.0, 23.7* | 0.01 |
| Single | -157.8 | -359.5, 43.9* | 0.01 |
| BMI at recruitment (kg/m^2^ ) | 12.4 | 3.5, 21.2** | 0.02 |
| Tobacco smoking during pregnancy | -163.3 | -298.7, -27.9** | 0.01 |
| Alcohol consumption during pregnancy | -93.2 | -327.3, 140.8 | 0.00 |
| Recreational drug consumption during pregnancy | -435.8 | -880.7, 9.1 | 0.01 |
| Infection during pregnancy | -293.1 | -474.6, -111.5** | 0.02 |
| Previous low birth weight (< 2500g) infant in multipara ( n= 235) | -190.9 | -399.3, 17.5* | 0.01 |
| Previous preterm infant in multipara (n = 235) | -236.9 | -505.1, 31.3* | 0.01 |
| Inter pregnancy interval^ǂ^  low (< 18 months)  ≥ 18 and ≤ 60 months  large (> 60 months) | -84.3  reference  -23.1 | -246.9, 78.3  -229.1, 182.8 | 0.00 |
| **Early pregnancy levels of** |  |  |  |
| Cadmium (ng/mL) | -82.7 | -150.44. -14.9** | 0.01 |
| Manganese (ng/mL) | 7.1 | -7.81; 21.9 | 0.00 |
| HgT (ng/mL) | -3.6 | -75.73; 68..6 | 0.00 |
| Lead (ng/mL) | -8.50 | -17.36; 0.35* | 0.01 |
| sum of PCBs (ngg of lipid) | 173.93 | -32.03; 379.90* | 0.01 |

Abbreviations: SD, standard deviation;

* *p*-value< 0.25, ** p-value < 0.05*** p-value < 0.0001

ǂ Time interval between the delivery date of the preceding pregnancy and the beginning date of GESTE pregnancy (obtained by ultrasound).

**Table S3.** Linear relations between birth weight corrected for gestational age and mothers’ plasma levels of PBDEs (non-transformed) in early pregnancy (N= 349)

| **PBDEs** | **Simple linear regression**± | | **Adjusted linear regression**# | | | |
| --- | --- | --- | --- | --- | --- | --- |
|  |  |  | **Model A** | | **Model B** | |
|  | **β** | **95% CI** | **β** | **95% CI** | **β** | **95% CI** |
| **PBDEs (ng/g of lipid)** |  |  |  |  |  |  |
| PBDE-47 | 0.5 | -0.4; 1.3 | 0.2 | -0.7; 1.1 | 0.1 | -0.9; 1.2 |
| PBDE-99 | 2.5 | -2.1; 7.3 | 1.0 | -3.7; 5.7 | 0.8 | -5.3; 6.8 |
| PBDE-100 | 3.2 | -2.45; 8.9 | 3.2 | -2.4; 8.8 | 1.9 | -5.2; 9.0 |
| PBDE-153 | -2.7 | -6.1; 0.6 | -2.4 | -5.7; 0.9 | -2.4 | -6.4; 1.7 |
| ΣPBDEs | 0.3 | -0.4; 0.9 | 0.1 | -0.6; 0.8 | 0.0 | -0.8; 0.9 |
|  |  |  |  |  |  |  |
| **PBDEs (ng/mL)** ^‡^ |  |  |  |  |  |  |
| PBDE-47 | 97.2 | -63.5; 257.9 | 40.5 | -127.0; 207.9 | 26.3 | -184.0; 236.7 |
| PBDE-99 | 619.5 | -273.8; 1512.7 | 328.8 | -587.6; 1245.1 | 321.5 | -877.7; 1520.6 |
| PBDE-100 | 566.2 | -489.4; 1621.9 | 568.3 | -467.4; 1603.9 | 451.6 | -973.7; 1876.9 |
| PBDE-153 | -440.3 | -1128.6; 248.1 | -400.7 | -1082.9; 281.5 | -420.2 | -1279.7; 439.2 |
| ΣPBDEs | 68.4 | -60.6; 197.3 | 26.9 | -106.9; 160.9 | 13.6 | -155.8; 182.9 |
|  |  |  |  |  |  |  |
| **PBDEs (ng/mL) adjusted for total plasma lipids** |  |  |  |  |  |  |
| PBDE-47 | 103.2 | -57.8; 264.2 | 43.7 | -123.9; 211.4 | 40.2 | -170.9; 251.3 |
| PBDE-99 | 740.6 | -168.7; 1649.9 | 423.2 | -511.1; 1357.6 | 517.7 | -710.2; 1745.6 |
| PBDE-100 | 546.8 | -509.4; 1603.1 | 546.4 | -491.2; 1583.9 | 477.8 | -946.2; 1901.8 |
| PBDE-153 | -375.5 | -1082.2; 331.1 | -350.9 | -1052.2; 350.3 | -282.6 | -1183.3; 618.1 |
| ΣPBDEs | 77.6 | -52.2; 207.3 | 33.2 | -101.4; 167.8 | 33.3 | -138.2; 205.1 |

Abbreviations: CI, confidence interval; PBDE(s), Polybrominated diphenyl ether(s); ΣPBDE, sum of BDE-47, 99, 100 and 153.

± Non-adjusted for potential confounders

# Adjusted for potential confounders

**Model A:** Adjusted on mother’s age at delivery, marital status, BMI at recruitment, infection, smoking status during pregnancy, ΣPCBs (CB-138, CB-153 and CB-180 expressed in ng/g of lipid), mothers total blood levels of lead (ng/ml),

**Model B:** Model A + previous pregnancies history (preterm infant and low birth weight infant) in multipara only (N= 234).

‡Total lipids were not taken into account in these models.


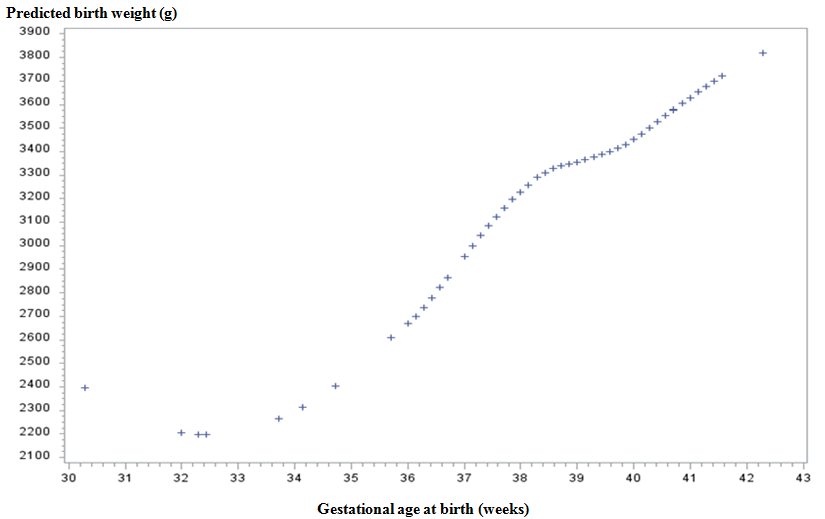


**Figure S1.** Relation between predicted BW (obtained with proc LOESS) and gestational age at birth
